# Bluefin tuna (*Thunnus thynnus*) larvae exploit rare food sources to break food limitations in their warm oligotrophic environment

**DOI:** 10.1093/plankt/fbaf006

**Published:** 2025-03-01

**Authors:** Patricia Reglero, Maria Pilar Tugores, Josefin Titelman, Mar Santandreu, Melissa Martin, Rosa Balbin, Diego Alvarez-Berastegui, Asvin P Torres, Nelly Calcina, Laura Leyva, Øyvind Fiksen

**Affiliations:** Instituto Español de Oceanografía-CSIC, Centre Oceanogràfic de les Balears, Muelle de Poniente s/n, 07015 Palma de Mallorca, Spain; Instituto Español de Oceanografía-CSIC, Centre Oceanogràfic de les Balears, Muelle de Poniente s/n, 07015 Palma de Mallorca, Spain; Department of Biosciences, University of Oslo, PO Box 1066 Blindern, 0316 Oslo, Norway; Instituto Español de Oceanografía-CSIC, Centre Oceanogràfic de les Balears, Muelle de Poniente s/n, 07015 Palma de Mallorca, Spain; Instituto Español de Oceanografía-CSIC, Centre Oceanogràfic de les Balears, Muelle de Poniente s/n, 07015 Palma de Mallorca, Spain; Instituto Español de Oceanografía-CSIC, Centre Oceanogràfic de les Balears, Muelle de Poniente s/n, 07015 Palma de Mallorca, Spain; Instituto Español de Oceanografía-CSIC, Centre Oceanogràfic de les Balears, Muelle de Poniente s/n, 07015 Palma de Mallorca, Spain; Instituto Español de Oceanografía-CSIC, Centre Oceanogràfic de les Balears, Muelle de Poniente s/n, 07015 Palma de Mallorca, Spain; Instituto Español de Oceanografía-CSIC, Centre Oceanogràfic de les Balears, Muelle de Poniente s/n, 07015 Palma de Mallorca, Spain; Instituto Español de Oceanografía-CSIC, Centre Oceanogràfic de les Balears, Muelle de Poniente s/n, 07015 Palma de Mallorca, Spain; Department of Biological Sciences, University of Bergen, PO Box 7803, 5020 Bergen, Norway

**Keywords:** starvation, nauplii, cladocera, copepods, temperature, fish larvae, *Thunnus thynnus*

## Abstract

Tuna spawns in some of the warmest and most oligotrophic areas worldwide. At the same time, starvation is often considered the main source of mortality for fish larvae. Here we assess if plankton availability is sufficient to sustain the high growth potential of tuna (*Thunnus thynnus*) larvae in a major spawning ground in the warm oligotrophic Mediterranean Sea. We combine field data with a model of larval foraging, growth, and bioenergetics and find that just enough food is available in the warm surface layer to sustain the high growth rate of the larvae. For bluefin tuna, higher temperatures can be beneficial if prey abundance is high, 10 000–27 000 nauplii m^−3^, 14–36 cladocerans m^−3^, 3–7 copepods m^−3^, but critical if not. While nauplii alone may not sustain the growth potential of even the smallest tuna larvae, our model predicts that including some larger copepods or cladocerans in the diet reduces food limitation and can sustain growth even in the warmest years. The combination of clear Mediterranean waters and the occasional copepod or cladocerans alleviates food limitation despite the low zooplankton concentrations in the area. In conclusion, oligotrophic spawning areas allow for fast growth of these foraging efficient larvae, unless temperatures exceed 28°C.

## INTRODUCTION

The interplay between temperature and resource acquisition is seminal for the growth and development of all organisms in all environments. Also, for some long-lived species, and especially those that exploit a vast range of environmental conditions, the interplay between somatic growth, ontogenetic development, temperature, and resource acquisition is intimately tied to their relatively complex life cycles (Visser *et al.*, 2020). Tuna fish are typical examples of the latter.

Tuna typically spawns in some of the warmest ocean temperatures worldwide and in strongly oligotrophic areas (Schaefer, 2001; Hiraoka *et al.*, 2022). This holds both for species inhabiting tropical warm waters (e.g. yellowfin tuna, *Thunnus albacares*, bigeye tuna, *Thunnus obesus*) and for species, such as the Atlantic bluefin tuna (*Thunnus thynnus*), that are well-adapted to cold waters with seasonally warmer periods (Schaefer, 2001; Reglero *et al.*, 2014). The reproductive strategies of tuna species and their tight connection to warm waters relate to the thermal tolerance and adaptations of eggs and larvae that require temperatures above 20°C for survival (Reglero *et al.*, 2018). The association of spawning to oligotrophic areas is less obvious. Temperature can shift growth rates from positive to negative depending on food availability (Huey and Kingsolver, 2019; Fiksen and Reglero, 2022). Therefore, larvae would do better if high temperatures were accompanied by high food availability. Why would species with some of the fastest-growing larvae in the ocean (Malca *et al.*, 2022) select some of the poorest areas in terms of food resources to reproduce? This question becomes especially intriguing when one considers the parental effort of migrating over 5 000 km from the North Atlantic to aggregate at some warm oligotrophic spatiotemporally restricted spawning ground located e.g. in the Mediterranean Sea.

While the Mediterranean Sea is the main spawning ground for the eastern stock of Atlantic bluefin tuna the dynamics in the Mediterranean Sea can also control the populations in the feeding grounds of the north and central Atlantic oceans (Lutcavage *et al.*, 1999; Galuardi *et al.*, 2010). Bluefin tuna born in the Western Mediterranean are also present in the spawning grounds in the Slope Sea in the Western Atlantic (Richardson *et al.*, 2016; Arregui *et al.*, 2018; Hernández *et al.*, 2022).

Large-scale long-term increasing trends in water temperature in the Mediterranean could potentially lead to higher recruitments in Atlantic bluefin tuna because larval growth increases with temperatures up to 30°C, given sufficient food (Reglero *et al.*, 2018). However, if food is limited then higher temperature increases metabolic costs and reduces growth, which can be detrimental to fish larvae (Fiksen and Reglero, 2022).

The Mediterranean is under massive anthropogenic multiple stress (Crise *et al.*, 2015). Climate, including increasing frequencies and amplitudes of marine heat waves (Garrabou *et al.*, 2019; Juza and Tintoré, 2021) may drive the system away from its historic steady state as an oligotrophic temperate sea (Polovina *et al.*, 2008; Lazzari *et al.*, 2014). It is therefore important to understand when food availability sustains high growth rates of the larvae in the warming waters of the Mediterranean Sea.

Atlantic bluefin tuna larvae are well adapted to find prey in the very clear Western Mediterranean offshore waters. At temperatures of 24°C, the development of large eyes, swimming capabilities and a stomach takes less than two weeks (Yúfera *et al.*, 2014). The rapid development allows for switching to a piscivorous diet during the post-flexion developmental stage (Blanco *et al.*, 2019). In turn, the dietary shift reduces potential food limitations relative to a pure plankton diet in the post-flexion larval stage (Reglero *et al.*, 2011; Blanco *et al.*, 2019). First-feeding tuna larvae feed mainly on copepod nauplii but also include some zooplankton in their diet (Llopiz and Hobday, 2015; Muhling *et al.*, 2017). In the Mediterranean Sea, the frequency of larger cladocerans and copepods in the diet increases with tuna larval ontogeny (Catalán *et al.*, 2011; Uriarte *et al.*, 2019; Shiroza *et al.*, 2022).

In the spawning ground in the Gulf of Mexico, starvation is predicted to be the most important mortality source for the early larvae (Shropshire *et al.*, 2022). However, the transport of prey from the more productive northeastern continental area in the Gulf of Mexico to the spawning grounds may reduce the food limitation (Kelly *et al.*, 2021; Shiroza *et al.*, 2022). Such mechanisms are not in place in the even more oligotrophic offshore waters of the Mediterranean Sea, where the spatial distribution of zooplankton prey is generally homogenous (Reglero *et al.*, 2017). To understand the occurrence of food limitations, the mechanisms behind food limitation, and how it affects habitat use we must integrate ontogenetic feeding capacities into models and combine these with size-structured time-series of plankton biomass estimates (Landry and Swalethorp, 2022; Malca *et al.*, 2022).

In Reglero *et al.* (2018) and Fiksen and Reglero (2022) we focused on the phenology of spawning in bluefin tuna and tried to answer the question of why Atlantic bluefin tuna spawn relatively early in the season compared to what is predicted from a purely temperature-driven physiology. Here we explore in depth if the plankton availability in the warm waters of the Mediterranean Sea is sufficient to sustain the high growth of the larvae, using a combination of our survey data and a simplified model of larval foraging and bioenergetics as in Fiksen and Reglero (2022). We describe and apply field observations of temperature, zooplankton prey, and bluefin tuna larvae obtained from oceanographic surveys at the major bluefin tuna spawning grounds in the Western Mediterranean. Using the model we examine how abundance and size-structure of zooplankton determine bluefin tuna growth in this Mediterranean spawning ground.

## MATERIAL AND METHODS

### Field data

We conducted two surveys from 24 June–6 July 2020 and 13–30 June 2022 onboard the R/V Ángeles Alvariño offshore in the Western Mediterranean Sea. The cruise targeted the peak time of reproduction and the main Atlantic bluefin tuna spawning area. In each station, hydrography was profiled using a CTD (SBE32). Temperatures in the entire mixed layer in both years were compared to the 20-year average temperatures in the area (IBAMAR database https://ibamardatabase.wordpress.com/metadata-exploration/).

**Fig. 1:**
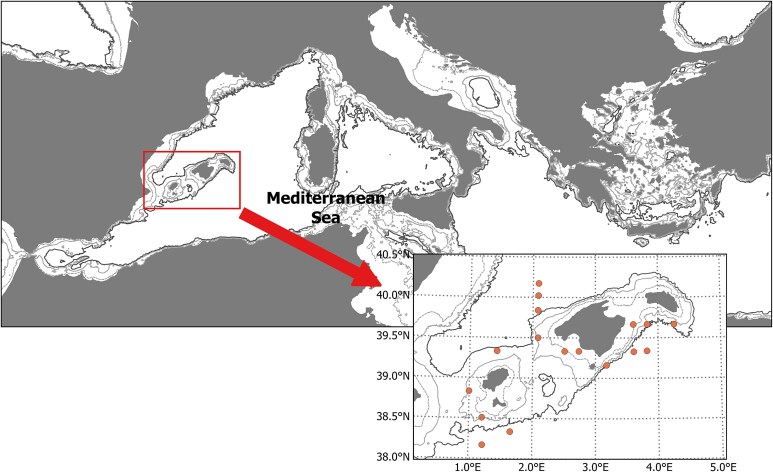
The study region is located around the Balearic Islands in the Western Mediterranean Sea. Circles indicate the stations where micro, mesozooplankton and bluefin tuna larval samples were counted.

We sampled bluefin tuna larvae using oblique hauls from 0 to 20 m depth using a Bongo net of 90 cm mouth diameter and a mesh size of 500 μm within a systematic grid of stations (Fig. 1). One replicate sample was preserved in 4% buffered formalin in seawater, and the second replicate in ethanol.

Meso- and microzooplankton were sampled with a Bongo net of 20 cm mouth diameter and mesh sizes of 200 and 53 μm, respectively. These Bongo nets were attached above the Bongo-90. The volume of water filtered was measured with flowmeters located at the centre of each net. In total 17 stations were sampled for meso- and microzooplankton abundance, such that the different water masses and depths were covered in the sampling. Upon retrieval, the nets were rinsed and microzooplankton samples were immediately preserved in 96% ethanol. Two aliquots of the mesozooplankton samples were separated whereupon one was immediately preserved in 96% ethanol and the other frozen at 20°C.

Nauplii in microzooplankton samples were counted under a dissecting microscope. We also measured the length of 191 nauplii selected randomly from 5 stations. The mesozooplankton samples were split with a Folsom splitter and subsamples were counted under a dissecting microscope considering a minimum of 400 individuals. The sorting was based on Rose (1933), Trégouboff and Rose (1978), Vives and Shmeleva (2010), and Vives *et al.* (2007). To estimate the total number nauplii, copepods and cladocera, the number of counted individuals was multiplied by the corresponding split factor and densities (# m^−3^) estimated by dividing the total number of individuals in each station by the filtered volume. Fish larvae from the Bongo-90 samples were sorted under a stereo microscope, and bluefin tuna larvae were identified and counted using determination keys for the area (Alemany, 1997). The larvae were photographed using a microscope with a camera and Image-pro software ® and measured for standard length (mm).

### Specific growth rates

We applied a bioenergetic model to estimate the larval growth rates at different prey densities based on Fiksen and Reglero (2022):


(1) \begin{equation*} {\mathrm{SGR}}_{\mathrm{T}, prey}\mathrm{W}=\mathrm{\alpha} {i}_{prey}-{\mathrm{R}}_{\mathrm{L},\mathrm{T}} \end{equation*}

where SGR_T,prey_ is the specific growth rate (mg dw mg^−1^ hour^1^), *W* is the larval dry weight (mg dw), α is the assimilation efficiency (= 0.77, see Fiksen and Reglero, 2022), i_prey_ is prey ingestion and *R* is the size- and temperature-dependent routine metabolism (both in mg dw h^−1^). *Ad-libitum* conditions in the laboratory were ensured by maintaining prey concentrations in the tanks at 10 rotifers ml^−1^ (see Fiksen and Reglero, 2022).

We apply a foraging model (see Fiksen and Reglero, 2022) to estimate ingestion of each type of prey that is readily available to tuna fish larvae of length L (m); nauplii, cladocerans, and copepods:


(2) \begin{equation*} {i}_{prey,L}={P}_{prey}{\beta}_{prey,L}\ {N}_{prey}\ {W}_{prey} \end{equation*}

where *P_prey_* is prey capture probability, ${i}_{prey,L}$ is the prey ingestion per hour of each prey type (the sum of all prey types is the total ingestion), *N_prey_* is the prey concentration (# m^−3^), *W_prey_* is the dry mass (in mg) of each prey type, and the search rate, β*_prey,L_* (m^3^ h^−1^)


**Fig. 2:**
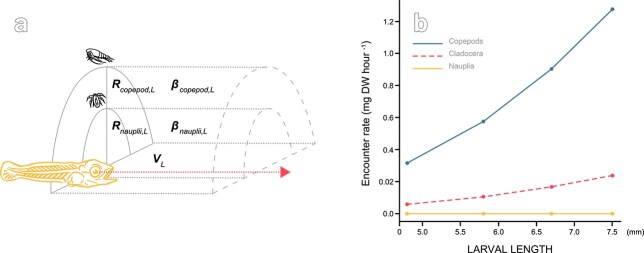
Conceptual figure of the modeled predator–prey encounters. **a**) The search rate, β*_prey,L_* (m^3^ h^−1^), depends on the prey detection distance *R_prey,L_* (m) which is larger for larger prey (copepods vs nauplii). The swimming velocity of the larva *V_L_*, is set to 3 body lengths s^−1^. The figure is adapted from Ottmann *et al.* (2022). **b**) Encounters of prey are higher for larger (copepods) than smaller prey (nauplii). Estimations are calculated for prey densities of 300 prey m^−3^ and temperature of 24.5°C.

(3) \begin{equation*} {\beta}_{prey,L=\frac{1}{2}\pi{R}_{prey,L}^2{V}_L} \end{equation*}

depends heavily on the prey detection distance *R_prey,L_* (m)—which is a function of prey size, light (day or night) and larval ontogeny (assumed proportional to body length) (Fig. 2a). Prey detection distance is sensitive to the visual acuity of the larval eye, which is related to the size of the eye and the density of photoreceptors at the retina both of which develop during ontogeny. The modeled visual acuity is based on measures of Southern bluefin tuna larvae through ontogeny (Hilder *et al.*, 2019). We assume the larva effectively scans half the visual field and primarily focuses upwards using the sea surface as background contrast to detect prey (Fig. 2a) resulting in higher encounters with larger than smaller prey (Fig. 2b). Swimming velocity of the larva *V_L_*, is set to 3 body lengths s^−1^. Capture probability *P_prey_* for nauplii is 1 for all larval sizes, and increases linearly from 0 to 1 for cladocerans and copepods as tuna larvae develop from first-feeding to post-flexion (as in Fiksen and Reglero, 2022; see also Catalán *et al.*, 2011, Fig. S1).

Energy expenditure (*R_L,T_*) is estimated as:


(4) \begin{align*} {R}_{L,T}=&\ 0.404{W}^{0.994}\ast 32\ast 0.88\ast \left(\frac{12}{34}\right) \nonumber \\ &\ast \left(\frac{100}{1000\ast 45}\right)\ast{Q_{10}}^{\frac{Temp-26}{10}} \end{align*}

where *R* increases almost proportionally to the size of the tuna larvae as $0.404{W}^{0.994}$ measured as μmol O_2_ h^−1^ at 26°C (Blanco *et al.*, 2020) and then converted to mg dry mass by applying factors for conversion to protein, carbon, and dry weight (($0.88\ast \left(\frac{12}{34}\right)\ast \left(\frac{100}{1000\ast 45}\right))$ factors in equation 4 and a Q_10_ = 2 for the dependency of metabolism with temperature (see Fiksen and Reglero, 2022 for more details on the growth and feeding processes).

### Simulations

First, we estimate daily specific growth rates at different prey densities and temperatures (${\mathrm{SGR}}_{\mathrm{T}, prey}$) and find the prey concentration where the maximum potential specific growth rate (SGR_T_, mg dw mg^−1^ day^−1^) was achieved. The temperature-dependent maximum potential specific growth rate, SGR_T_, (mg dw mg^−1^ day^−1^) was calculated based on results from temperature-controlled experiments at *ad libitum* conditions. SGR_T_ increases with temperature *T* (see Reglero *et al.*, 2018) as


(5) \begin{equation*} {SGR}_T=0.0418T-0.8355 \end{equation*}

**Fig. 3:**
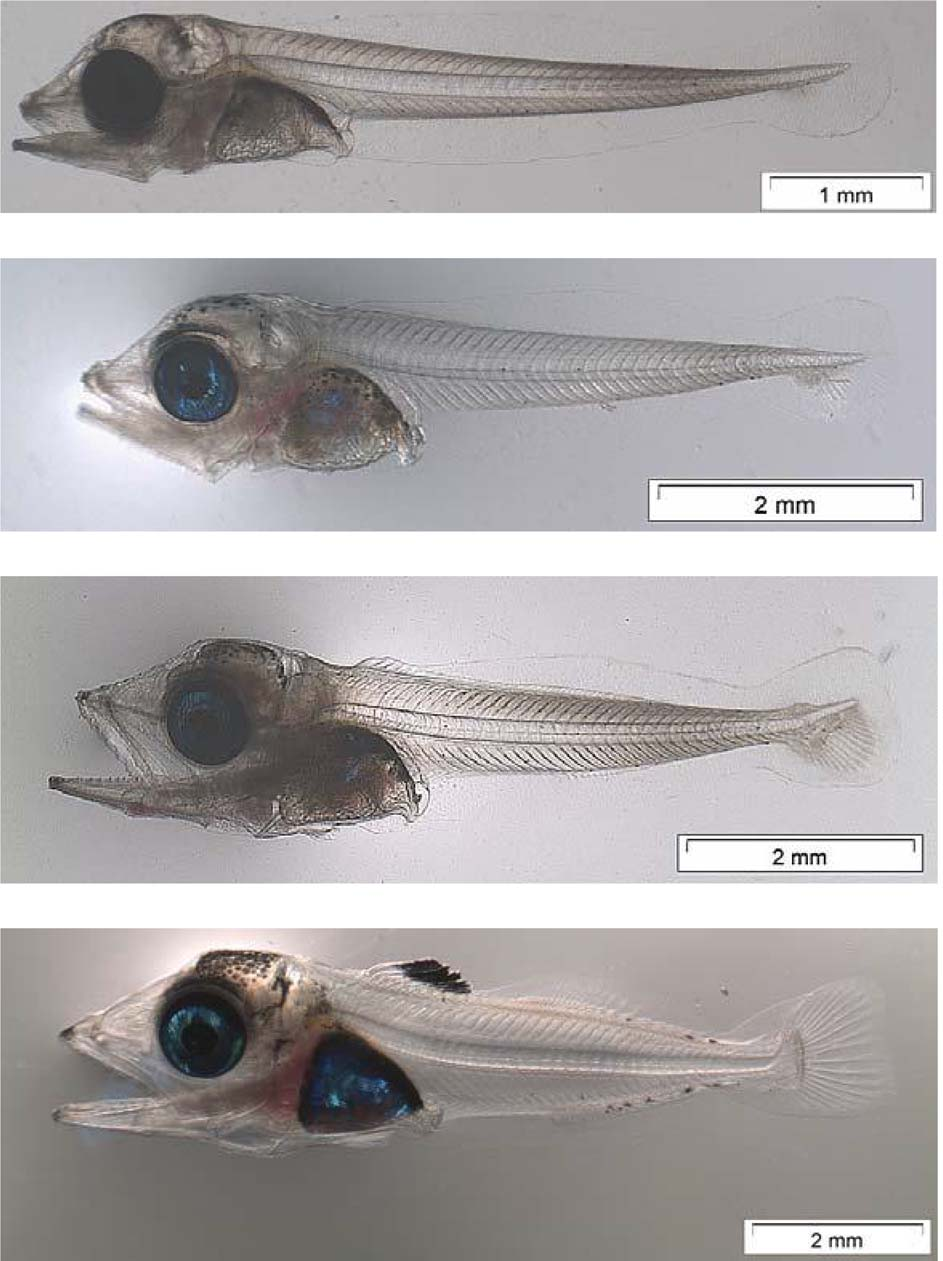
Atlantic bluefin tuna larval lengths, 4.8, 5.8, 6.7, and 7.5 mm, corresponding to stages pre-flexion (F0), first-caudal fin rays (F1), flexion (F2) and post-flexion (F3).

The larvae do not feed in darkness (personal observation, Catalan 2011, Blanco *et al.* 2017), therefore we exclude night time in the daily estimate of ingestion potential. Daylight hours in the temperature-controlled experiment were similar to natural photoperiod and set to 15 hours (Reglero *et al.*, 2018). We considered four larval lengths, 4.8, 5.8, 6.7 and 7.5 mm, corresponding to the stages pre-flexion (F0), first-caudal fin rays (F1), flexion (F2) and post-flexion (F3) respectively (Fig. 3, Blanco *et al.*, 2019). Larval dry weights (*W*, mg dw) were related to standard length (*SL*, mm) as (Reglero *et al.*, 2018):


(6) \begin{equation*} W=0.0008\ast{e}^{0.9052 SL} \end{equation*}

We first used a range of nauplii, cladoceran and copepod densities to estimate the growth rates at different prey densities until the larval maximum potential specific growth rates were achieved. These are the main prey items for tuna larvae in the Mediterranean Sea (Catalán *et al.*, 2011; Muhling *et al.*, 2017; Uriarte *et al.*, 2019; Shiroza *et al.*, 2022) The average length (mm) of the nauplii was 0.15 (±0.05 SD) (n = 191, this study) corresponding to a dry weight of 0.11 μg assuming the length-weight relationship in Berggreen *et al.* (1988). We set cladoceran length to 0.8 mm corresponding to 3.15 μg dry weight (Catalán *et al.*, 2007), and copepod length to 1 mm (own unpublished data) corresponding to 11.1 μg dry weight (Berggreen *et al.*, 1988). We also considered three different temperatures, 22, 25 and 28°C, based on the temperature range observed at the Balearic spawning areas. Finally, we assess if plankton concentrations estimated at surveys in the field is sufficient to sustain the high growth potential of tuna larvae in a major spawning ground in the Mediterranean Sea. Sensitivity analyses were run assuming naupliar lengths of 0.15 mm and weight of 0.11 μg as in this study and lengths of 0.3 mm and weight of 0.5 μg based on Catalán *et al.* (2007) (Fig. S2) as used in Fiksen and Reglero (2022).

## RESULTS

### Prey abundance in the field

**Fig. 4:**
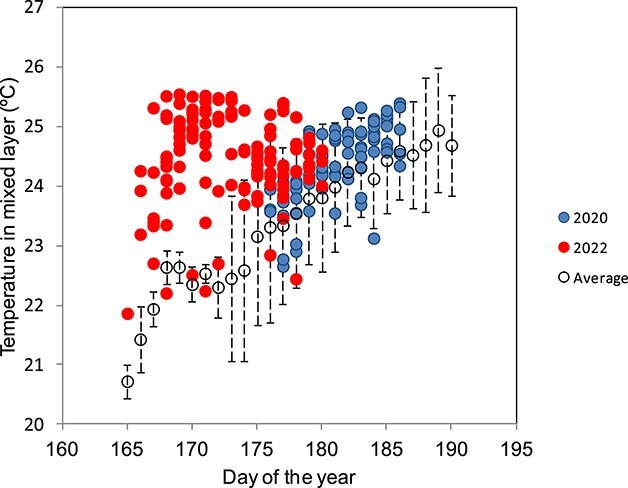
Timeline of field data temperatures in the mixed layer. Each dot represents one observation of temperature measured in one station for 2020, 2022 and the 20-year average ± SD for every day of the year estimated using temperature observations for stations sampled in yearly cruises.

The water column was strongly stratified during both years. Temperatures in the entire mixed layer (0-30 m) during 2022 exceeded the 20-year average by up to 3–4°C, whereas temperatures in 2020 were mostly within the normal range (Fig. 4). Temperatures in the mixed layer in the 17 stations sampled for micro and mesozooplankton ranged between 23.1–25.3°C in 2020 and 23.9–25.5°C in 2022 with averages of 24.4 (±0.7 SD) and 24.6 (±0.5 SD) respectively. Nauplii, copepod, cladocera and bluefin tuna larval abundances were unrelated to temperature both years (p > 0.05 all analyses).

**Fig. 5:**
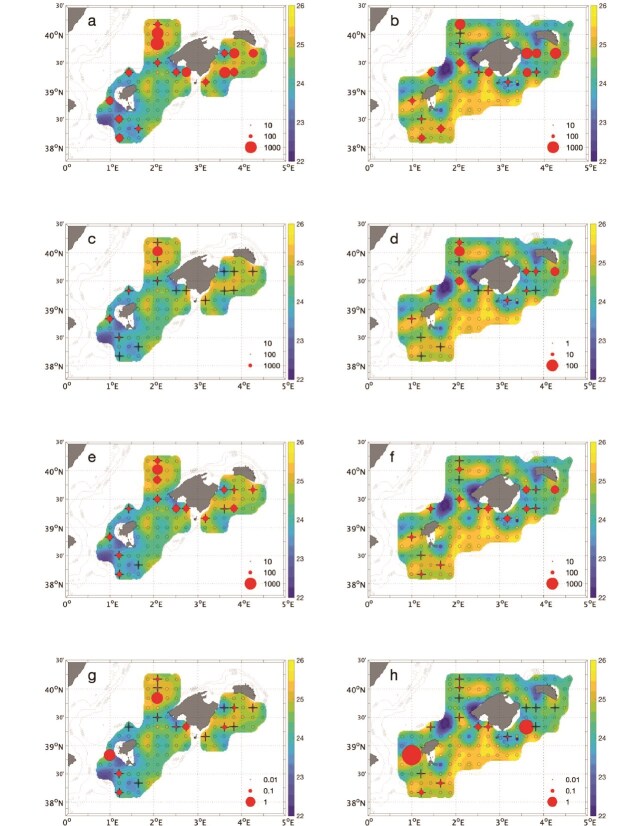
Spatial distribution of temperature in the mixed layer (°C) and abundances (# m^−3^, dots) of nauplii ((**a**) 2020 (left) and **b**) 2022 (right)), cladocera (note different scale in 2020 and 2022 (**c**) 2020 (left) and **d**) 2022 (right)), copepods (**e**) 2020 (left) and **f**) 2022 (right)), and bluefin tuna larvae (note scale in bubbles for abundances are log-transformed, **g**) 2020 (left) and **h**) 2022 (right)). Empty circles indicate CTD casts.

Nauplii and copepod abundances were similar in 2020 and 2022 (Table 1; Fig. 5a, b; Fig. 5e, f). Cladocera abundances were higher in 2020 than 2022 (Table 1; Fig. 5c, d). No cladocera were found in 3 and 1 stations in 2020 and 2022 respectively (Fig. 5c,d). Bluefin tuna abundance were lower in 2020 than 2022 (Table 1; Fig. 5g, h). No bluefin tuna larvae were found in 3 and 2 stations in 2020 and 2022 respectively. Distributions of bluefin tuna larvae were unrelated to prey distributions during both years (*P* > 0.05).

**Table 1:** Nauplii, cladocera, copepod and bluefin tuna abundances sampled in 2020 and 2022

	2020		2022	
	Range (m^−3^)	Average (± SD)	Range (m^−3^)	Average (± SD)
Nauplii	169–1 244	506 (±303)	43–918	311 (±438)
Cladocera	1–7 272	653 ((±1834)	1–74	17 (± 22)
Copepod	26–773	258 (±173)	3–494	154 (± 126)
Bluefin	0.002–1.7	0.27 (±0.52)	0.005–15.28	1.16 (±3.7)

### Model predictions

**Fig. 6:**
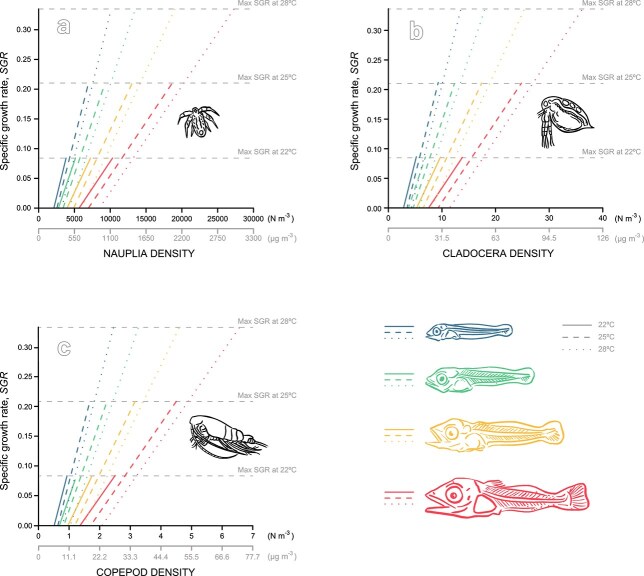
Food limitation on tuna daylight specific growth rates at different temperatures, 22°C, 25°C and 28°C at different developmental stages (larva F0: blue; larva F1: green; larva F2: yellow and larva F3: red) feeding only on **a**) nauplii, **b**) cladocerans and **c**) copepods. The type of line reflects the different temperatures, straight line 22°C, dashed line 25°C, dotted line 28°C. Horizontal gray dashed lines are SGR libitum at the different temperatures 22°C, 25°C, 28°C.

When the diet consisted only of nauplii, prey abundance limits the growth of tuna with a stronger effect on larger tuna larvae (Fig. 6a). The nauplii prey densities required for maintaining maximum growth rates at 22°C were in the order of 4 000 m^−3^ (development stage F0), 5 000 m^−3^ (F1), 7 000 m^−3^ (F2), and 10 000 m^−3^ (F3) (Fig. 6a, Table S1). Such nauplii abundances are at least an order of magnitude higher than that observed in the field (Fig. 5a,b). Standard metabolic expenses could only be supported by nauplii concentrations much higher than those in the field, eg. in the order of 2000 m^−3^ (development stage F0), 2 800 m^−3^ (F1), 3 900 m^−3^ (F2) and 5 600 m^−3^ (F3) at 22°C (Fig. 6a, Table S1). Prey density becomes more critical for maintenance and growth as temperatures increase (Fig. 6a). Nauplii prey densities required for growing at maximum growth rates at 25°C were in the order of 7 000 m^−3^ (development stage F0), 9 000 m^−3^ (F1), 13 000 m^−3^ (F2), and 19 000 m^−3^ (F3), and even higher at 28°C, in the order of 10 000 m^−3^ (development stage F0), 14 000 m^−3^ (F1), 19 000 m^−3^ (F2), and 27 000 m^−3^ (F3) (Fig. 6a, Table S1).

When the diet consisted only of cladocerans, the prey density to sustain growth was three orders of magnitude lower when compared to when feeding only nauplii (Fig. 6b, Table S1). The cladoceran prey densities required for maintaining maximum growth rates at 22°C were ca. 5 m^−3^ (development stage F0), 7 m^−3^ (F1), 10 m^−3^ (F2), and 14 m^−3^ (F3). The cladocera abundances (nm^−3^) that sustain maximum growth rates increase linearly with increasing temperatures (Fig. 6b, Table S1). Such cladocera abundances are within the order of magnitude of abundances observed in the field (Fig. 3c, d). Including copepods benefited larvae of all sizes and supplied the required energy to grow at maximum growth rates (Fig. 6c, Table S1).

Sensitivity analyses showed that food limitation was prevented at higher nauplii abundances assuming naupliar lengths of 0.15 mm and weight of 0.11 μg as in this study (Fig. S2) and lengths of 0.3 mm and weight of 0.5 μg based on Catalán *et al.* (2007) (Fig. S2) as used in Fiksen and Reglero (2022).

The relative benefit of including the larger copepods and cladocerans in the diet compared to the nauplii-only scenario is clear in the field simulation (Fig. S3). Larval growth is limited in most locations if feeding only on nauplii both in 2020 (Fig. S3a) and 2022 (Fig. S3b). Feeding only on cladocerans could sustain maximum growth rates at all larval sizes in 2020 (Fig. S3c), but not always in 2022 (Fig. S3d). In 2022 the spatial distribution of cladocera was patchy, and cladocerans were absent in some stations (Fig. 6d) when compared to the more homogeneous distribution in 2020 (Fig. 6c). A diet based on copepods prevented limitation and allowed for maximum specific growth rates for all developmental stages during both years (Fig. S3e, f). Feeding on a combination of all prey allowed maximum growth rates for all larval sizes in both years (Fig. S3g, h).

## DISCUSSION

While nauplii alone may not sustain the growth of even the smallest tuna larvae, our model predicts that supplementing the diet with larger zooplankton is necessary to break food limitations in the oligotrophic spawning grounds. When including cladocerans and copepods in the diet the model predicts that there is just enough food available in the warm surface layer to sustain the high growth and allow for the short planktivorous stage durations of the first-feeding tuna larvae. This is simply because, from the viewpoint of early-stage tuna larvae, food availability appears higher than one would expect in an oligotrophic sea. We did not include density-dependent competition for food. The high swimming speed, clear water, presence of some relatively large prey, and rapid development of visual acuity contribute to large clearance rates and sustained growth.

Tuna larvae are well-adapted to take advantage of the clear water and bright optical conditions of the Mediterranean. Already early in life, bluefin tuna develop eyes that allow for an acute vision for prey detection (Hilder *et al.*, 2019). Prey encounter for larvae is further optimized by the spawning occurring during the season with the most hours of daylight (Gordoa *et al.*, 2009) and during full moon (Ottmann *et al.*, 2023), and with the highest abundance of cladocerans (de Puelles *et al.*, 2014).

The notion of apparent food limitation, compared to the real food limitation may be explained by a combination of physiological adaptations to the clear oligotrophic waters. Naupliar concentrations in the Mediterranean (ca 400, this study) are almost an order of magnitude lower than in more eutrophic coastal waters (e.g. Titelman and Fiksen, 2004). However, the optical conditions are substantially better in the Mediterranean. Assuming a tuna larva detects a nauplius at 3 cm in the clear Mediterranean, while only at 1 cm in darker North Atlantic coastal waters means 9 times difference in search volume and required prey concentration for the same ingestion rate. Spawning in clear waters facilitates growth as long as digestive capacity does not constrain food uptake (Ljungström *et al.*, 2020), and predators are sfew. In regions with high planktivore fish densities, the benefit in prey encounter rates is quickly outweighed by high predation risk.

For bluefin tuna, higher temperatures can be beneficial if prey abundance is high, but critical if not. Our study emphasizes the need to consider the interplay between temperature and food. A likely scenario is that food becomes limited with increasing temperatures, for example, during a marine heatwave (Fig. 4). This could, for example, explain the annual seasonality of bluefin tuna offspring survival (Fiksen and Reglero, 2022). During the marine heatwave in 2022, temperatures exceeded average temperatures, which increased larval metabolic rates. However, marine heatwaves do not influence the optical conditions and therefore do not change the ability of tuna to detect their prey.

Feeding rates in our model are constrained by mechanisms driving visual prey encounters and larval capture success highlighting the role of prey size. The model could be extended to include the interaction between ingestion, specific dynamic action and assimilation efficiency, as well as their temperature dependences (Peck and Daewel, 2007). Metabolism increases as temperature rises. This leads to more energy gain only if the higher temperature speeds up digestion, more than it causes energy loss (Ljungström *et al.*, 2020).We could also consider the gut as a state variable that limits ingestion when full or restricts growth rate when empty (Fiksen and Reglero, 2022). Although our model is simple, it effectively predicts that plankton availability is sufficient to sustain maximum growth rates.

The model does not explicitly account for the energy expenditures associated with activity and swimming. At higher temperatures, larval swimming may be faster, but energy demands also increase (Moyano *et al.*, 2016). When prey abounds, larvae can afford to decrease swimming speeds and reduce both energetic costs and their predation risk (Mahjoub *et al.*, 2011; Fouzai *et al.*, 2019). Although experimental data on the behavior of larvae is still limited (De La Gándara *et al.*, 2016), the food limitation index developed here considers both encounter and metabolism and could thus be applied to explore behaviors further.

In the model, we characterized prey quality by their energy content and size. While copepods are more abundant than cladocerans (de Puelles *et al.*, 2014), the larger cladocerans with darkly pigmented eyes are probably easier to detect visually. These two prey types also differ in stoichiometry (Andersen and Hessen, 1991). Cladocerans have a relatively higher N content than copepods and are also richer in phosphorous (P) (Andersen and Hessen, 1991; Gismervik, 1997). While the benefit of N and protein is obvious for bodybuilding, P is also important for developing and maintaining both skeletons and muscles. Fatty acid acquisition in the retina improves vision and the development of neural tissues, both of which are essential for foraging behavior in bluefin tuna larvae (Koven *et al.*, 2018) and many other fish (Mourente and Tocher, 2009). While DHA is an essential fatty acid, it cannot be synthesized by the larvae and must be obtained from the diet (Masuda, 2003). Sudden changes in water temperature and prey can alter fatty acid profiles which may result in an extremely low percentage of DHA (Hiraoka *et al.*, 2022). We hypothesize that feeding on prey with pigmented eyes, such as cladocerans and copepods of the family Corycaeidae *(*e.g. *Farranula and Corycaeus),* that often abound in the diet (Catalán *et al.* 2011; Uriarte *et al.*, 2019; Shiroza *et al.*, 2022), may benefit vision. Without good vision, the tuna larvae could not make a living at the low zooplankton concentrations in the Mediterranean.

Nauplii are rarely sampled in the Mediterranean. In both years of our study, the nauplii densities of ca. 400 m^−3^ were much lower than in coastal more productive areas of the Norwest Mediterranean, where abundances are around 10 000 m^−3^ (Calbet, 2001). In coastal areas of Mallorca, concentrations are also on the lower end, around 1 000 m^−3^ (de Puelles *et al.*, 2014). While we only sampled down to the thermocline, i.e. within the habitat of the tuna larvae, Calbet (2001) sampled from the surface down to ca. 60–70 m where the deep chlorophyll maximum (DCM) is found. The DCM is typically associated with the highest abundances of zooplankton in these waters. However, the DCM is located below the thermocline and as such inaccessible for the larval bluefin tuna that cannot survive in the cold waters at the DCM. Tuna larvae only have access to the zooplankton in the upper 30 m, within the upper mixed layer, where temperatures are warm. Therefore, quantifying tuna's prey fields at the relevant depths remains essential.

While food is required for larval growth, it is not the key factor for bluefin tuna when selecting their fine-scale habitats within the spawning area. Nauplii and temperature in the Northwestern Mediterranean spawning area are spatially relatively uniform, and especially temperature is mostly related to the time of the year (this study, Reglero *et al.*, 2012). In Atlantic bluefin tuna larvae in the Gulf of Mexico, starvation is the major source of mortality in the younger larvae and survival is greatest in mesoscale eddies that form on the shelf where food is more abundant than in the open sea (Shropshire *et al.*, 2022). The mesoscale eddies in the Mediterranean spawning area do not work in the same way. While they favor larval retention (Díaz-Barroso *et al.*, 2022), food abundance within them is similar to that outside the structures (this study). We instead hypothesize that the retention areas ensure efficient piscivorous feeding after postflexion. Such a strategy is well-suited to overcome the planktivorous food limitation that arises in later larval stages, and also speed up growth (Reglero *et al.*, 2011; Blanco *et al.*, 2019).

## CONCLUSIONS

Understanding Atlantic bluefin tuna in the Mediterranean Sea also benefits insight into other large migratory species in other systems, such as tropical tuna. Typically, spawning strategies and seasonal cycles are driven by temperature and resource availability (Visser *et al.*, 2020). While a warmer climate is the new norm, and marine heat wave scenarios add temperature-related stress to the ecosystem, particularly in the Mediterranean (Garrabou *et al.*, 2022), warming does not significantly alter the optical environment at least if not accompanied by excess runoff (e.g. Kaartvedt and Titelman 2018). The importance of the optical environment has generally been overlooked for tuna and similar species. In many ways the low prey abundances are balanced out by the great optical conditions, and by supplementing the numerically abundant nauplii with an occasional copepod or cladocerans, such that from the eyes of a tuna larva the clear oligotrophic Mediterranean waters may not be food limited after all.

## Supplementary Material

Reglero_etal_2024_JPlanktonResearch_Supplementary_revision_notracks_fbaf006

## Data Availability

Data and code are available through the Github Repository: https://github.com/MPilar123/BFT_Feeding_items/blob/main/Software_MS_Bluefin%20tuna%20larvae%20exploit%20rare%20food%20sources%20to%20break%20food%20limitations%20in%20their%20warm%20oligotrophic%20environment_Author%20MP%20Tugores_v2024.r.
