# Supplementary material for: Bluefin tuna (Thunnus thynnus) larvae exploit rare food sources to break food limitations in their warm oligotrophic environment
Source: J Plankton Res. 2025 Mar 1;47(2):fbaf006. doi: 10.1093/plankt/fbaf006 (PMC11879179; doi:10.1093/plankt/fbaf006)
Supplement: Reglero_etal_2024_JPlanktonResearch_Supplementary_revision_notracks_fbaf006 [file reglero_etal_2024_jplanktonresearch_supplementary_revision_notracks_fbaf006.docx]

**Supplementary material**

**Bluefin tuna (*Thunnus thynnus*) larvae exploit rare food sources to break food limitations in their warm oligotrophic environment**

Authors: Patricia Reglero^1*^, Maria Pilar Tugores^1^, Josefin Titelman^2^, Mar Santandreu^1^, Melissa Martin^1^, Rosa Balbin^1^, Diego Alvarez-Berastegui^1^, Asvin Perez-Torres^1^, Nelly Lisbeth Calcina^1^, Laura Leyva^1^, Øyvind Fiksen^3^

^1^ Instituto Español de Oceanografía-CSIC, Centre Oceanogràfic de les Balears, 07015 Palma de Mallorca, Spain.

^2^ Department of Biosciences, PO box 1066 Blindern, 0316 Oslo, Norway

^3^ Department of Biological Sciences, University of Bergen, 5020 Bergen, Norway.

*Corresponding author: Patricia Reglero, patricia.reglero@ieo.csic.es

**Table S1**

**Food limitation on tuna larval growth at different temperatures**. Minimum (Min) nauplia, cladocera and copepods density (**(#** m^-3^)) required for bluefin tuna larvae not to starve and just cover standard metabolic needs**.** Maximum (Max) nauplia, cladocera and copepods density (**(#** m^-3^)) at which bluefin tuna larvae achieve the maximum theoretical growth, as computed in the laboratory at libitum at 22ºC, 25ºC and 28ºC at different developmental stages (larva F0: 4.8 mm; larva F1: 5.8 mm; larva F2: 6.7 mm and larva F3: 7.5 mm).

|  |  | **Nauplia density**  **(#** m^-3^) | | **Cladocera density**  **(#** m^-3^) | | **Copepod density**  **(#** m^-3^) | |
| --- | --- | --- | --- | --- | --- | --- | --- |
| **Temperature** | **SL (**mm) | **Min** | **Max** | **Min** | **Max** | **Min** | **Max** |
| 22 ºC | 4.8 | 2,129 | 3,865 | 3 | 5 | 0.5 | 1 |
|  | 5.8 | 2,826 | 5,143 | 4 | 7 | 1 | 1 |
|  | 6.7 | 3,970 | 7,240 | 5 | 10 | 1 | 2 |
|  | 7.5 | 5,646 | 10,318 | 8 | 14 | 1 | 3 |
| 25 ºC | 4.8 | 2,621 | 6,946 | 4 | 9 | 1 | 2 |
|  | 5.8 | 3,479 | 9,251 | 5 | 12 | 1 | 2 |
|  | 6.7 | 4,887 | 13,034 | 7 | 17 | 1 | 3 |
|  | 7.5 | 6,952 | 18,590 | 9 | 25 | 2 | 5 |
| 28 ºC | 4.8 | 3,227 | 10,140 | 4 | 14 | 1 | 3 |
|  | 5.8 | 4,283 | 13,509 | 6 | 18 | 1 | 3 |
|  | 6.7 | 6,017 | 19,040 | 8 | 25 | 1 | 5 |
|  | 7.5 | 8,558 | 27,163 | 11 | 36 | 2 | 7 |

**Figure S1. Relationship between tuna larval dry weight (mg) and capture probability when feeding on copepods and cladocera.** Dots indicate different the average weight for the four developmental stages considered in the model, F0, F1, F2 and F3 and the larval size at hatching (when capture probability is zero) and the maximum weigth at post-flexion (when capture probability is one). The SD for the average weight (mg) as measured in the laboratory for each developmental stage is ±0.03 for F0, ± 0.08 for F1, ±0.10 for F2 and ± 0.43 for F3 as measured in Blanco et al. 2019.

**Figure S2. Food limitation on tuna larval growth at different temperatures**, 28ºC, 25ºC and 22ºC at different developmental stages (larva F0: blue; larva F1: green; larva F2: yellow and larva F3: red) feeding only on nauplii assuming a nauplia length of 0.15 mm and weight of 0.11 µg this study (Fig S1a) and length of 0.3 mm and weight of 0.5 µg based on Catalán et al. 2007 (Fig S1b) as used in Fiksen and Reglero (2021). Tones reflect the different temperatures, the darker the tone the highest temperature. Horizontal dashed lines are SGR libitum at the different temperatures 22ºC (light grey), 25ºC (grey), 28ºC (black).


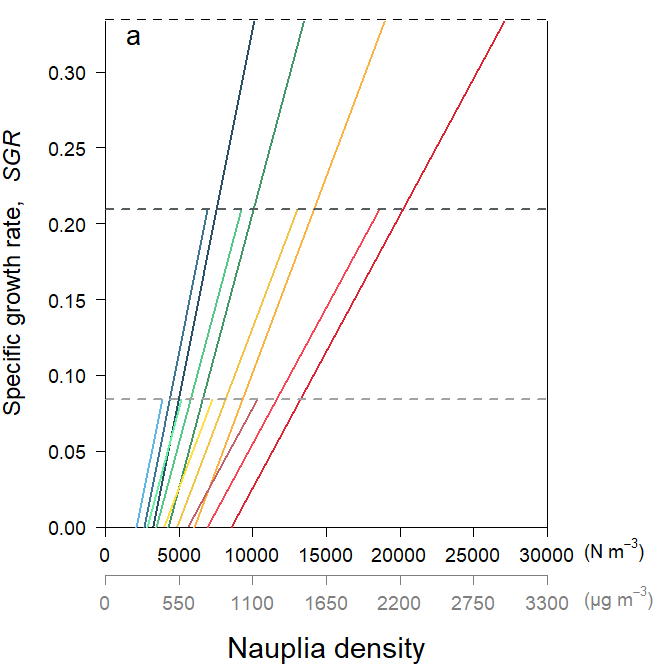

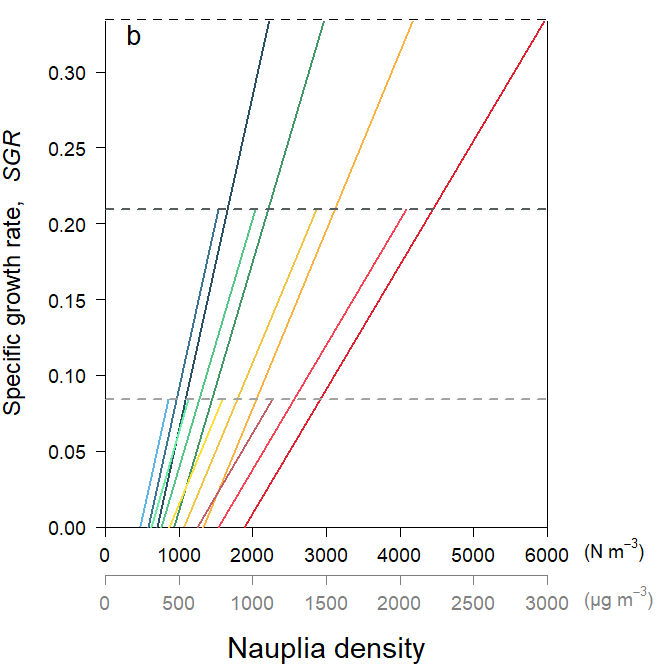


**Figure S3**. Specific growth rates at field prey densities and temperatures, at different developmental stages (larva F0: blue; larva F1: green; larva F2: yellow and larva F3: red) feeding only on nauplii (a,b), cladocerans (c,d) and copepods (e,f) and all prey (g,h) in 2020 and 2022. Maximum bar length indicates SGR libitum.

2020

2022


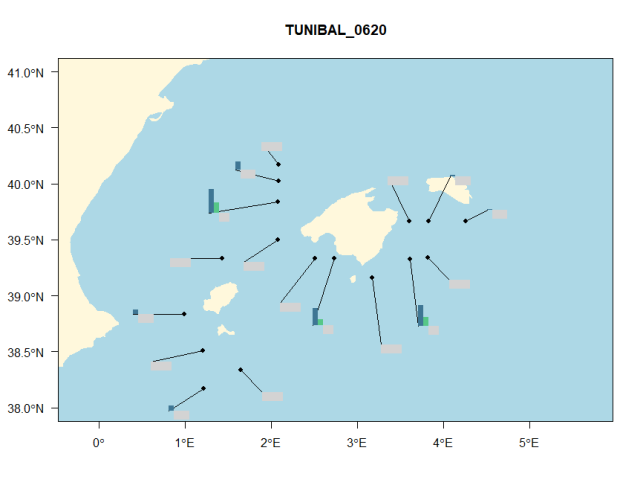

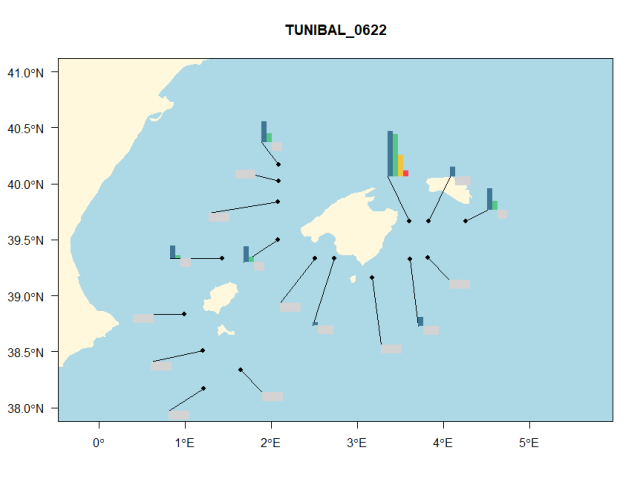


b

a


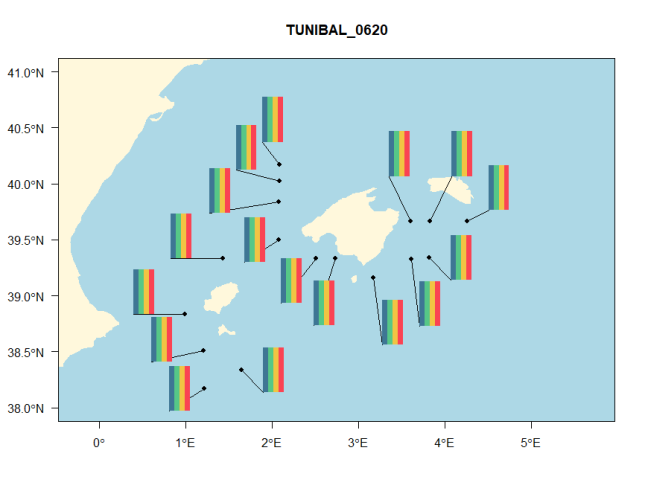

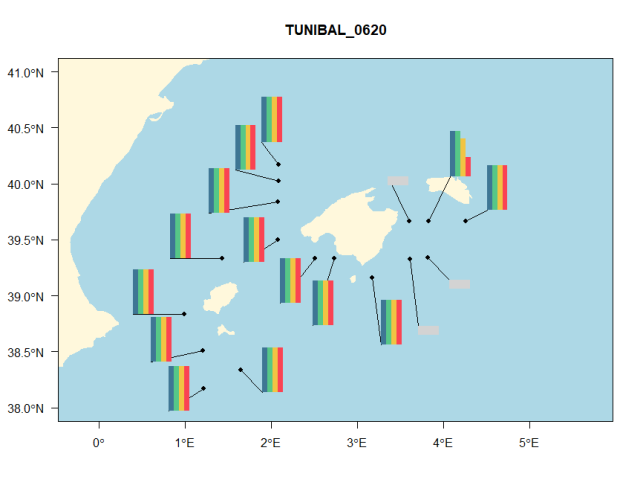


d

c


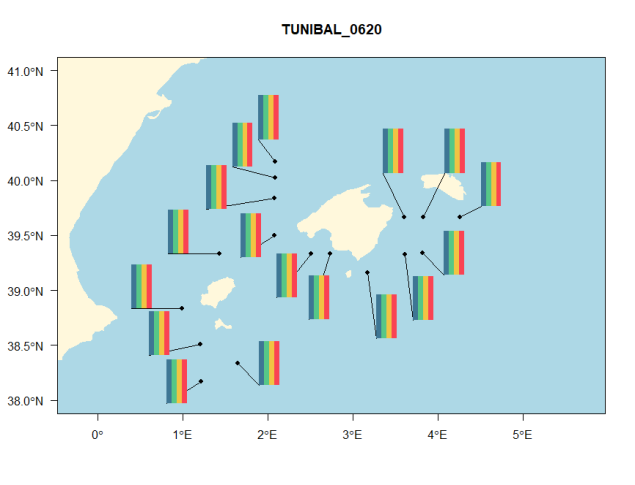

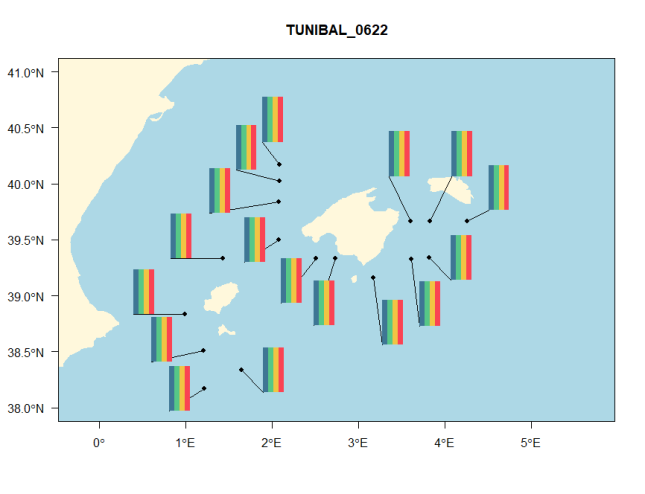


f

e


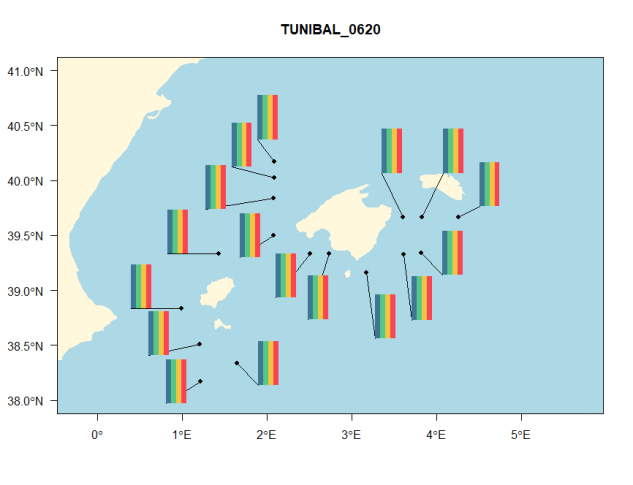

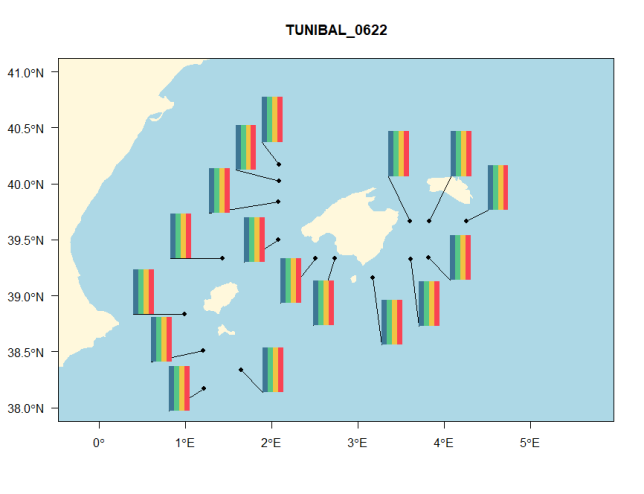


h

g

**References**

Blanco, E., Reglero, P., Hernández De Rojas, A., Ortega, A., De La Gándara, F., and Folkvord, A. (2019) The effect of nutritional condition by two nucleic acid derived indices on the growth to post-flexion of Atlantic bluefin tuna and Atlantic bonito larvae. Journal of Experimental Marine Biology and Ecology, 519, 151182.
